# Body Packing and Its Radiologic Manifestations: A Review Article

**DOI:** 10.5812/iranjradiol.4757

**Published:** 2011-12-25

**Authors:** Makhtoom Shahnazi, Morteza Sanei Taheri, Ramin Pourghorban

**Affiliations:** 1 Department of Radiology, Loghman Hakim Hospital, Shahid Beheshti University of Medical Sciences, Tehran, Iran; 2 Department of Radiology, Shohada-E-Tajrish Hospital, Shahid Beheshti University of Medical Sciences, Tehran, Iran

**Keywords:** Pelvis, Abdomen, Radiography, Abdominal, Tomography, X-Ray Computed

## Abstract

Body packing is described as using the abdominal or pelvic cavity for concealing illegal drugs. Leakage from the packets may cause catastrophic effects on smugglers and medical history is not reliable in these patients. Moreover, new sophisticated smuggling techniques make it imperative that radiologists and emergency physicians understand and familiarize themselves with the different radiological manifestations of ingested drug packets. Currently, there is no gold standard for imaging patients suspected of body packing; nevertheless, computed tomography (CT) seems to be the best modality for packet detection and unenhanced CT without bowel preparation is a reliable technique for detection of ingested packets. On abdominal radiography, packets may be visualized as oval or round radiopaque foreign bodies surrounded by a gas halo. In the literature, sensitivity of abdominal radiography is reported from 74% to 100%. Visualization of the drug packets may be strikingly hampered by administration of oral or intravenous contrast medium in abdomino-pelvic CT; hence, contrast-enhanced CT does not seem to be a suitable modality for searching the ingested packets in suspicious smugglers.

## 1. Introduction

Body packing was first described by Dr. Deitel and Dr. Syed in 1973. They found a patient with small bowel obstruction 13 days after swallowing a condom containing hashish. The small bowel was emptied preoperatively by a long-tube, and the impacted bolus was removed by enterotomy [1]. Since then, drug smuggling by internal concealment is a well-recognized mode of transporting illegal drugs. Moreover, body packing has increased since September 11, 2001, possibly due to increased border security which has made conventional trafficking more difficult [2].

**Figure 1:**
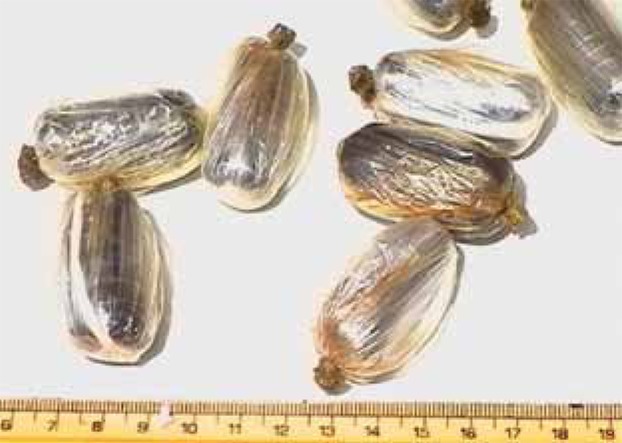
Illicit drugs evacuated from a body packer. They are packed tightly and wrapped into aluminum foil.

Body packers may also be called “swallowers”, “couriers”, “internal carriers”, or “mules” whereas the term “body stuffing” refers to the swallowing of relatively small amounts of loosely wrapped drug because of the fear of arrest and without intended attempt to transport the drugs across borders [3]. Traub et al. reported the first two cases of pediatric body packing [4]. Moreover, Cordero and colleagues reported a pregnant cocaine body packer who required a perimortem cesarean section after the rupture of a cocaine packet [5]. Body packers may smuggle a wide range of illicit drugs including cocaine, heroin, amphetamines, methylenedioxymethamphetamine (“ecstasy”), marijuana and hashish [6][7][8][9]. They smuggle illicit drugs mainly by swallowing. However, insertions of packets into the rectum and vagina for concealment have also been reported [1]. Drugs are usually packed tightly and wrapped into a sheath like finger of latex gloves, plastic bags, condom, aluminum foil (Figure 1) or balloon [10][11]. After swallowing the drugs, constipating agents such as diphenoxylate or loperamide may be used to prolong the transit time. The transit times may vary from one day to three weeks [12]. After entering the destination site, the smugglers might use laxatives to help pass their packets [13].

## 2. Clinical Presentation

Body packers are referred with three types of presentations including acute symptoms of drug toxicity, bowel obstruction, or medical examination after detention or arrest by law-enforcement officers [14][15][16].

A detailed history, if possible, should be obtained to uncover the secret of body packing. Body packers are often unreliable historians. In addition, history taking might be unsuccessful because of their inability to cooperate due to profound drug-induced toxic effects [17]. Physical examination should include mental status, vital signs, pupil size, bowel motion and skin findings. Physicians should be familiar with different clinical manifestations of drug toxicity. Opioid toxicity presents with a depressed level of consciousness. Opiate toxicity should be suspected when the clinical triad of pupillary miosis, central nervous system (CNS) depression and respiratory depression is present. Cocaine overdose causes euphoria, anxiety, behavioral change, acute toxic psychosis, muscle rigidity, mydriasis, fever, diaphoresis, tachycardia and hypertension followed by seizures and cardiovascular collapse [18].

Body stuffers are more likely to present with symptoms of toxicity due to poor packaging of the drug. Furthermore, leakage from the packets before they rupture may cause impending catastrophic effects [19]. As a result, careful physical examination related to illicit drugs and surveillance should be performed in suspected body packers.

## 3. Imaging Assessment

### 3.1. Abdominal X-Ray

Diagnostic imaging is a valuable technique in clinical management of poisoned patients presenting to emergency rooms in a comatose state. As brain imaging shows different presentations of acute intoxication [20], abdominal imaging may help the physician to detect body packers and prevent catastrophic effects on smugglers.

**Figure 2:**
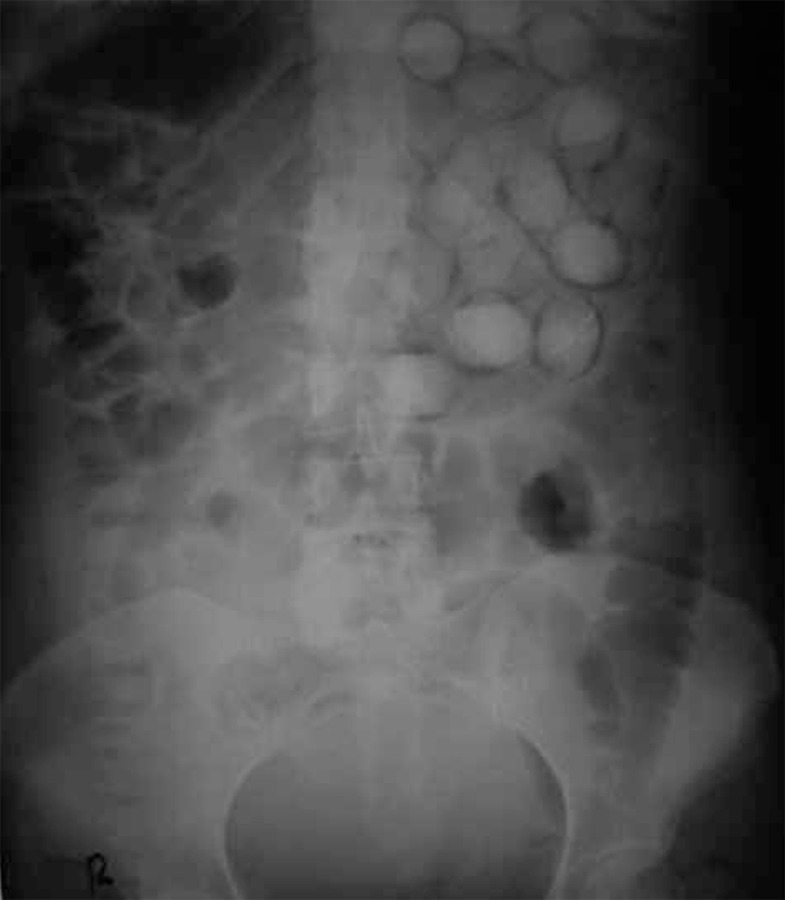
Abdominal x-ray (the same patient as Figure 1) reveals multiple, oval radiopaque packets throughout the abdomen.

**Figure 3:**
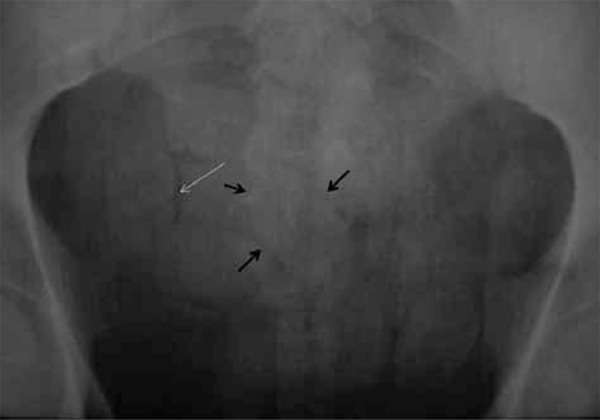
Pelvic x-ray demonstrates multiple radiopaque foreign bodies. Double-condom sign (black arrows), as air trapped between layers of latex and rosette-like appearance (white arrow), made by air trapped in condom knot are depicted.

Radiological evaluation is an effective method for diagnosis of body packers in suspected drug carriers. Low price and high availability make plain abdominal x-ray a relatively good screening tool in evaluating suspected body packers. In the literature, the sensitivity of plain abdominal x-ray has been reported from 74% to 100% [17][21][22][23][24]. However, such a high sensitivity may be too optimistic since a negative radiograph often results in the suspect’s release. Several imaging findings on abdominal x-ray are indicative of body packing, such as multiple radio-dense foreign bodies (Figure 2) or “double-condom” sign [25] (Figure 3) in which a crescent of air trapped between the different layers of latex makes them visible. In a retrospective analysis of the plain abdominal films of 53 “body packers”, Beerman et al. described the presence of the “double-condom sign” in the proximal ascending colon or upper gastrointestinal tract; a key imaging finding in the diagnosis of cocaine smugglers [26]. “Rosette like appearance” (Figure 3) is formed by air trapped in the knot where a condom is tied [15][22][26]. Nevertheless, radiological findings of packets may depend on the size, number, density and air-substance interfaces.

McCarron and Wood [27] described three different types of packages, with the following physical and radiographic characteristics and risks for rupture:

Type 1: Condoms or fingers of latex gloves contained cocaine in loose white powder form. The package material was stuffed with cocaine, folded back on itself and tied again at the opposite end. The first type of packages appeared as well-defined circular or cigar-shaped white opacities on plain films. Ties, if apparent, had a rosette like pattern. This form of packaging was most prone to be broken.

Type 2: Five to seven layers of latex covered the cocaine with small ties on each side. They were relatively large and uniform in size. These radiopaque bundles were oblong. On abdominal images, gas halos were seen with no apparent ties. This type was less prone to leaching as compared to the former one.

Type 3: Hardened cocaine was wrapped in both aluminum foil and tubular latex associated with some ties at both ends. On abdominal x-ray, they did not demonstrate as foreign bodies. Leaching or breaking of cocaine was not appreciated in type 3 packaging.

False-positive results in plain abdominal study may be due to large urinary bladder stones, other causes of intra-abdominal calcifications or inspissated stool, especially if the patient has taken constipating agents deliberately, as mentioned before. False-negative findings may be the result of the reader’s lack of experience in interpretation of plain films or poor quality of the study [22][28]. In a series of 82 cases admitted for abdominal x-ray, Karhunen et al. encountered nine (11.0%) true positives, three (3.6%) false positives, and one (1.2%) false negative [22].

Moreover, nowadays body packers use more sophisticated smuggling techniques by using aluminum foil, plastic food wrap or carbon paper to reduce the radio density of ingested drug packets. These new clever smuggling methods cause even more false-negative results [29].

### 3.2. Ultrasonography

Few studies have proposed that ultrasound represents a relatively valuable diagnostic method in the assessment of ingested drug packages. Alzen et al. reported that ultrasound correctly found the position of the “body-pack” in 20 out of 24 exams as compared to plain x-ray results [30]. Hierholzer et al. found that both abdominal x-ray and ultrasound correctly identified seven of 12 individuals who had ingested drug packages. In five of the 12 individuals, ultrasound as well as abdominal x-ray were correctly unremarkable with regard to abdominal foreign bodies [31]. However, till now there are not enough data to support the use of this tool in the evaluation of body packers.

### 3.3. Contrast-Enhanced Radiography

**Figure 4:**
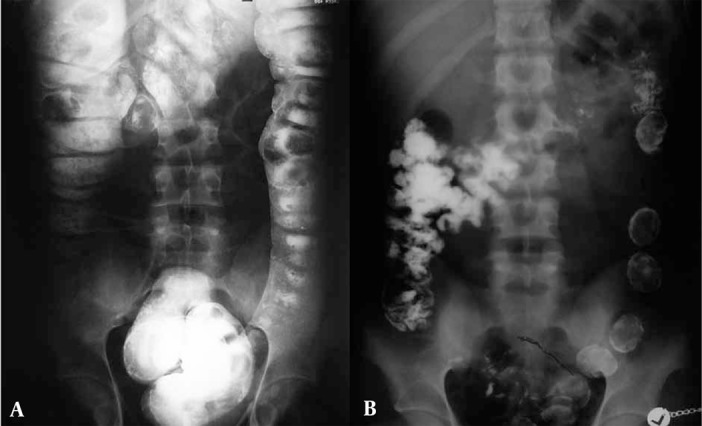
A, Barium enema was performed in a suspected body packer. Notice that there is no suggestion of abnormal foreign bodies in the descending colon; B, Post-evacuation image clearly shows the barium coated packets.

Contrast-enhanced radiography depicts drug packets as filling defects in the contrast medium. However, contrast medium can also influence the visualization of the packets (Figure 4). Marc et al. proposed oral administration of 60 mL of water-soluble contrast medium (amidotrizoate + meglumine) for medical management of cocaine body-packers after the initial drug detection in the urine. They reported that the sensitivity of this method ranged from 91.7 to 100% during the first 3 days and both false positive and false negative rates were about 4 percent [23].

### 3.4. CT Scan Without Contrast

**Figure 5:**
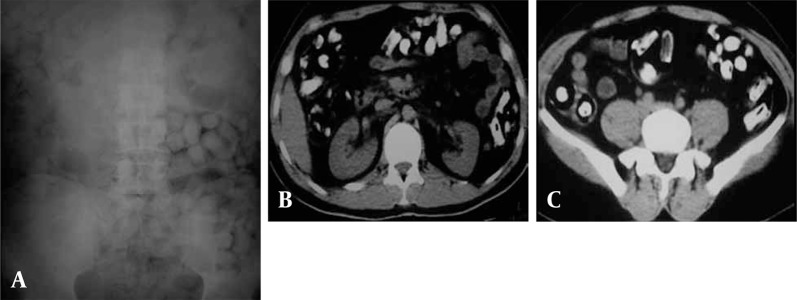
A, Abdominopelvic plain x-ray reveals several uniform radiopaque packets; B-C, Abdominal CT scan without oral contrast shows numerous randomly distributed packets within the small bowel and colon. Their density is 150-170HU which is compatible with the density of opium.

**Figure 6:**
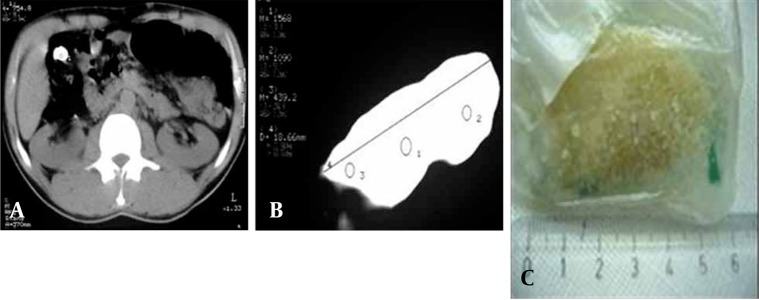
A, Abdominal CT scan without contrast shows a crack packet with the density of more than +1000 HU and length of 6 cm in the hepatic flexure; B, The packet is extracted and evaluated outside the body by CT. The density of the packet is still more than +1000 HU; C, The length of the extracted packet is about 6 centimeters.

**Figure 7:**
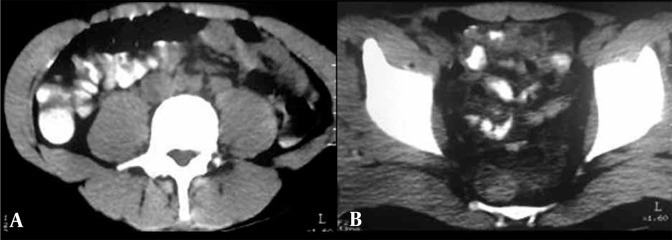
A-B, Non-contrast CT shows hyperdense material mixed with bowel contents with no history of prior barium or other contrast study. The findings are consistent with opened opium packets.

Abdominal computed tomography (CT) shows the body packets as multiple, oval or round and somehow uniform radiodense foreign bodies scattered throughout the abdomen (Figure 5 and 6). An incomplete hyperdense rim around the packets and a mixture of high density with normal bowel contents (Figure 7) are indicative of ruptured packets [32].

Eng et al. described a case of a body stuffer after ingesting a large packet containing multiple small packets with a falsely negative non-contrast enhanced CT [33].

The authors conducted a study to determine the sensitivity of abdominal CT scan without contrast in determining the presence, number and location of opium packets. In 12 cases who ingested opium packets, we found that CT scan without contrast can clearly identify the packets in all cases and no false negative cases were found. In addition, the density of the packets was assessed. The mean of minimum Hounsfield Unit (HU) was 163.8 ± 19.6 and the mean of maximum HU was 205.3 ± 32.8. The authors concluded that abdominal CT scan without contrast may be a suitable modality in identifying opium packets in suspicious cases [15][34].

CT has also been used experimentally to determine the contents of packets on the basis of differences in the HU: cocaine had a value of -219 HU and heroin had a value of -520 HU [35].

Yang and colleagues evaluated 158 cases of suspected drug packers with abdominal CT without oral or intravenous contrast medium. The content of the evacuated packets was analyzed chemically. Among them, 124 cases were finally diagnosed as heroin body packers. Abdominal CT without contrast medium easily identified all cases of true body packers and established 100% sensitivity, 100% specificity, 100% positive predictive value (PPV) and 100% negative predictive value (NPV). For the 42 suspected body packers that underwent both CT and x-ray imaging, 29 cases were positive on plain abdominal radiography and 13 were negative. There were two cases with negative abdominal x-ray film results and positive CT results for heroin body packing. They reported these two cases as false-negative with plain x-ray after the existence of an evacuated drug packet was established chemically. They concluded that negative screen on plain film does not exclude body packing and if the results of abdominal plain x-ray remain equivocal in strongly suspicious cases, conventional abdominal CT may provide a more definitive answer [36]. Currently, there is no gold standard for imaging patients suspected of body packing; nevertheless, CT seems to be the best modality for packet detection and unenhanced CT without bowel preparation is a reliable technique for detection of ingested packets.

### 3.5. CT Scan With Oral Contrast

Hahn et al. reported one false-negative case seen on a helical abdominal CT scan with oral contrast. The patient in their study claimed that 50 out of 55 ingested packets were evacuated after whole bowel irrigation. Helical abdominal CT scan with oral contrast failed to show any residual packets; whereas, plain abdominal x-ray depicted the last packet. This packet ultimately passed with continued whole bowel irrigation [37]. The visualization of the drug packets may be influenced by administration of oral or intravenous contrast medium in the abdominopelvic CT. In addition, contrast medium strikingly hinders the visualization of ruptured packets, if any; hence, contrast-enhanced CT does not seem to be a suitable modality for searching the ingested packets in suspicious smugglers.

## 4. Discussion

Body packing may cause catastrophic effects on smugglers as a result of packet rupture. Being familiar with radiologic features of body packing is essential for radiologists and emergency physicians in improving the diagnostic accuracy and preventing hazardous complications. Plain and contrast-enhanced radiographs, CT with or without oral contrast agent and ultrasound are various radiologic modalities used for detection of illicit drugs in body packers. Among them, plain radiography and non-enhanced CT are being used more frequently in the literature in order to detect ingested packets in suspicious smugglers.
